# Interobserver variability in target volume delineation in definitive radiotherapy for thoracic esophageal cancer: a multi-center study from China

**DOI:** 10.1186/s13014-020-01691-4

**Published:** 2021-06-09

**Authors:** Xiao Chang, Wei Deng, Xin Wang, Zongmei Zhou, Jun Yang, Wenling Guo, Miaoling Liu, Xiaolu Qi, Ling Li, Kaixian Zhang, Min Zhang, Yonggang Shi, Ke Liu, Yidian Zhao, Huitao Wang, Zhilong Yu, Jihong Zhang, Lihua Wang, Xueying Qiao, Chun Han, Shuchai Zhu, Ruohui Zhang, Junqiang Chen, Cairong Hu, Fuquan Zhang, Xiaorong Hou, Qingsong Pang, Wencheng Zhang, Gaofeng Li, Hailei Lin, Xinchen Sun, Xiaolin Ge, Caihong Li, Hong Ge, Dingjie Li, Yadi Wang, Na Lu, Xianshu Gao, Shangbin Qin, Yuan Tian, Zefen Xiao

**Affiliations:** 1 Department of Radiation Oncology, National Cancer Center/National Clinical Research Center for Cancer/Cancer Hospital, Chinese Academy of Medical Sciences, Peking Union Medical College, 17 South Panjiayuan Lane, Beijing, 100021 People’s Republic of China; 2 Department of Oncology, First Affiliated Hospital of Xinxiang Medical University, 88 Jiankang Road, Weihui, 453100 Henan People’s Republic of China; 3 Department of Radiation Oncology, Affiliated Hospital of Hebei University, Baoding, 071000 People’s Republic of China; 4 Department of Oncology, Tengzhou Central People’s Hospital, Tengzhou, 277599 People’s Republic of China; 5 Department of Radiation Oncology, The First Affiliated Hospital of Zhengzhou University, Henan Cancer Hospital, Zhengzhou, Henan People’s Republic of China; 6 Department of Radiation Oncology, Anyang Tumor Hospital, The Fourth Affiliated Hospital of Henan University of Science and Technology, Anyang, 455000 People’s Republic of China; 7 Department of Radiation Oncology, The Affiliated Hospital of Inner Mongolia Medical University, Tongdao Road, Hohhot, 10050 Inner Mongolia People’s Republic of China; 8 Department of Radiation Oncology, The Fourth Hospital of Hebei Medical University, Shijiazhuang, 050011 People’s Republic of China; 9 Department of Radiation Oncology, Fujian Cancer Hospital/Fujian Medical University Cancer Hospital, Fuzhou, 350014 People’s Republic of China; 10 Department of Radiation Oncology, Peking Union Medical College Hospital, Chinese Academy of Medical Sciences, Peking Union Medical College, No. 1 Shuaifuyuan, Wangfujing Dongcheng District, Beijing, People’s Republic of China; 11 Department of Radiation Oncology, Tianjin Medical University Cancer Institute and Hospital/National Clinical Research Center for Cancer, Tianjin, 300060 People’s Republic of China; 12 Department of Radiation Oncology, Beijing Hospital, National Center of Gerontology, Beijing, 100730 People’s Republic of China; 13 Department of Radiation Oncology, The First Affiliated Hospital of Nanjing Medical University, Nanjing, 210029 People’s Republic of China; 14 Department of Radiation Oncology, Affiliated Cancer Hospital of Zhengzhou University, Zhengzhou, Henan 450008 People’s Republic of China; 15 Department of Radiation Oncology, The Seventh Medical Center of PLA General Hospital, Beijing, 100700 People’s Republic of China; 16 Department of Radiation Oncology, Peking University First Hospital, Peking University, Beijing, People’s Republic of China

**Keywords:** Cancer of the esophagus, Radiation therapy, Target volume delineation, Interobserver variability

## Abstract

**Purpose:**

To investigate the interobserver variability (IOV) in target volume delineation of definitive radiotherapy for thoracic esophageal cancer (TEC) among cancer centers in China, and ultimately improve contouring consistency as much as possible to lay the foundation for multi-center prospective studies.

**Methods:**

Sixteen cancer centers throughout China participated in this study. In Phase 1, three suitable cases with upper, middle, and lower TEC were chosen, and participants were asked to contour a group of gross tumor volume (GTV-T), nodal gross tumor volume (GTV-N) and clinical target volume (CTV) for each case based on their routine experience. In Phase 2, the same clinicians were instructed to follow a contouring protocol to re-contour another group of target volume. The variation of the target volume was analyzed and quantified using dice similarity coefficient (DSC).

**Results:**

Sixteen clinicians provided routine volumes, whereas ten provided both routine and protocol volumes for each case. The IOV of routine GTV-N was the most striking in all cases, with the smallest DSC of 0.37 (95% CI 0.32–0.42), followed by CTV, whereas GTV-T showed high consistency. After following the protocol, the smallest DSC of GTV-N was improved to 0.64 (95% CI 0.45–0.83, *P* = 0.005) but the DSC of GTV-T and CTV remained constant in most cases.

**Conclusion:**

Variability in target volume delineation was observed, but it could be significantly reduced and controlled using mandatory interventions.

**Supplementary information:**

**Supplementary information** accompanies this paper at 10.1186/s13014-020-01691-4.

## Introduction

Definitive radiotherapy (dRT) concurrent with chemotherapy has been recognized as standard treatment for patients with locally advanced or unresectable thoracic esophageal cancer [1], and accurate target volume delineation was a prerequisite for three-dimensional conformal and intensity-modulated radiotherapy (IMRT) techniques, especially when using simultaneous-integrated boost (SIB) radiotherapy to deliver a boost dose to the gross tumor volume (GTV-T) and nodal gross tumor volume (GTV-N) [2, 3]. In 1998, Tai et al. [4] observed interobserver variability (IOV) of target volume delineation in cervical esophageal cancer among 48 radiation oncologists, and the same team further discovered that the variation could be controlled with the help of special training [5]. However, the delineation variation in dRT for thoracic esophageal cancer has not been evaluated.

Traditionally, definitive radiotherapy (dRT) field borders for esophageal cancer were designated by 3–5-cm expansions proximally and distally beyond the primary lesion along the esophagus, based on 2-dimensional planning [6, 7]. Recently, based on the intensity modulated radiation therapy (IMRT) technique, an expert consensus on contouring guidelines [8] compiled by radiation oncologists from cancer centers throughout the United States was published, which recommends that the CTV should include the GTV and GTV-N with at least 1-cm margin in all directions. In China, there are presently no consensus reference contouring guidelines, and hence, some variance in dRT field is likely among different cancer centers. An investigation to address IOV in target volume delineation seemed appropriate, as the IOV appeared to have an impact on clinical outcomes in multi-center studies and could potentially be minimized with refined consensus guidelines [9, 10].

This study aimed to investigate the IOV in target volume delineation in dRT for thoracic esophageal cancer among cancer centers in China, and ultimately improve contouring consistency as much as possible to lay the foundation for the multi-center prospective study.

## Materials and methods

### Patients

The following clinical examinations of three cases were completed in the primary center: The barium meal films and esophagogastroduodenoscopy (EGD) helped in locating the site and length of the tumor; further, endoscopic ultrasound (EUS) and computed tomography (CT) were mainly used to determine invasive depth and the relationship of surrounding tissues. Besides, nodal status was comprehensively judged by EUS, CT, and 18-fluorodeoxyglucose-positron emission tomography computed tomography (PET/CT). Brain magnetic resonance imaging and PET/CT were performed to exclude distant metastasis.

Case 1: The primary lesion was in the upper thoracic esophagus, and its boundary to the surrounding tissue was unclear with suspicion of tracheal invasion. Suspicious lymph nodes were in Station 1R, 1L, 2L, 4L, and 5 [11].

Case 2: The primary lesion was in the middle thoracic esophagus with a limited range of suspected lymphatic metastasis in Station 2R and 4R.

Case 3: The primary lesion was in the lower thoracic esophagus with a wide range of lymphatic metastasis. Suspicious lymph nodes were in Station 2R, 8L, and near the course of the left gastric artery.

### Study layout

**Fig. 1:**
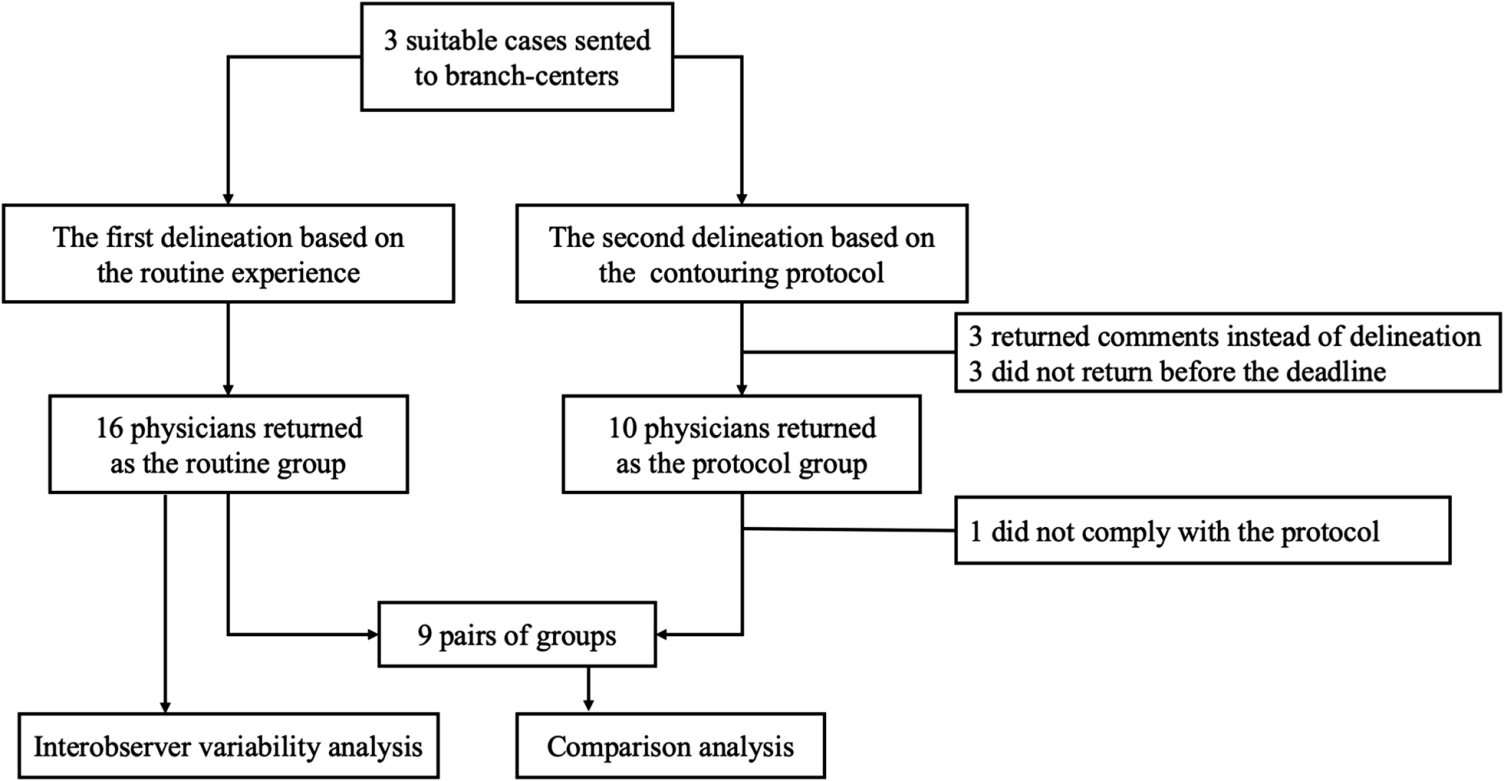
Flow chart of the study

The invited radiation oncologists from sixteen cancer centers were members of the Jing-Jin-Ji Esophageal and Esophagogastric Cancer Radiotherapy Oncology Group (3JECROG). A flow chart giving an overview of the study is shown in Fig. 1. In Phase 1, all branch centers received patient history, clinical examinations and planning CT fused with planning PET (slice thickness: 3.0 mm), and heads of the radiotherapy department were asked to identify their specialists in thoracic oncology to delineate the first group of GTV-T, GTV-N, and clinical target volume (CTV) based on their own routine experience, which was sent back to the primary center after completion and recorded as the routine group (RG). In Phase 2, differences and consistency of these target volumes between the centers were fully discussed at the second 3JECROG annual conference, and finally the contouring protocol was drafted and referential target volumes (RTVs) were drawn based on the expert opinions. Then, RTVs along with the contouring protocol (Additional file 1: The contouring protocol for guiding the determination of target volumes) and an atlas for target volume delineation [12] were sent to each center. The same specialists were asked to and give their opinions on RTVs and follow the protocol to re-delineate the second set of target volumes, which was recorded as the protocol group (PG).

### Contour analysis

**Fig. 2:**
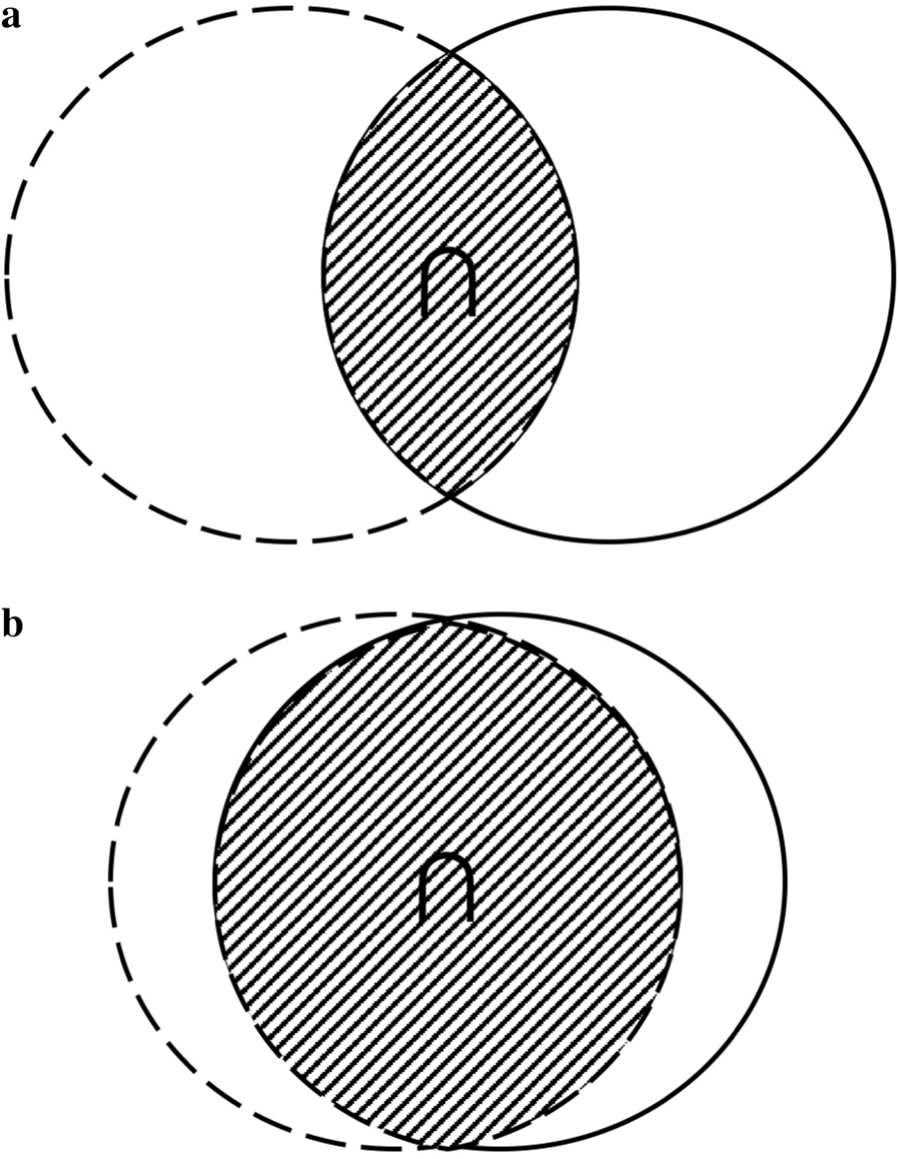
Contouring variability in spatial location evaluated by dice similarity coefficient (DSC). Sample “ ∩ ” with gray region represents overlapping region. Panels A and B are examples of poor and good spatial consistency, respectively

We introduced the dice similarity coefficient (DSC) [13] as a direct measure of the degree of target volume matching (Fig. 2), which had the ability to comprehensively evaluate the similarity in both volume and location. The method was used to calculate the spatial overlap between RTVs and target volumes from branch-centers. The value of DSC varies from 0 (completely disjoined) to 1 (absolutely overlapped). DSC was defined as follows:

$${\text{DSC}} = \frac{{2{\text{*V}}_{{({\text{RTVs}} \cap {\text{branch}})}} }}{{{\text{V}}_{{({\text{branch}})}} + {\text{V}}_{{({\text{RTVs}})}} }}$$

where V_(RTVs)_, V_(branch)_, and V _(RTVs∩branch)_ are the volume of RTVs, target volumes from branch-centers, and their overlapping region, respectively.

### Statistical analysis

The Shapiro–Wilk test [14] was used to check the distribution of continuous data for normality. The intergroup differences with normal distribution were evaluated using the paired t test; the ones with skewed distribution were evaluated using the rank test. All tests were two-sided, and a *P* < 0.05 was considered to indicate statistical significance. All statistical analyses were performed using R, version 3.5.1 (https://www.r-project.org/).

## Results

### Number of datasets received

**Fig. 3:**
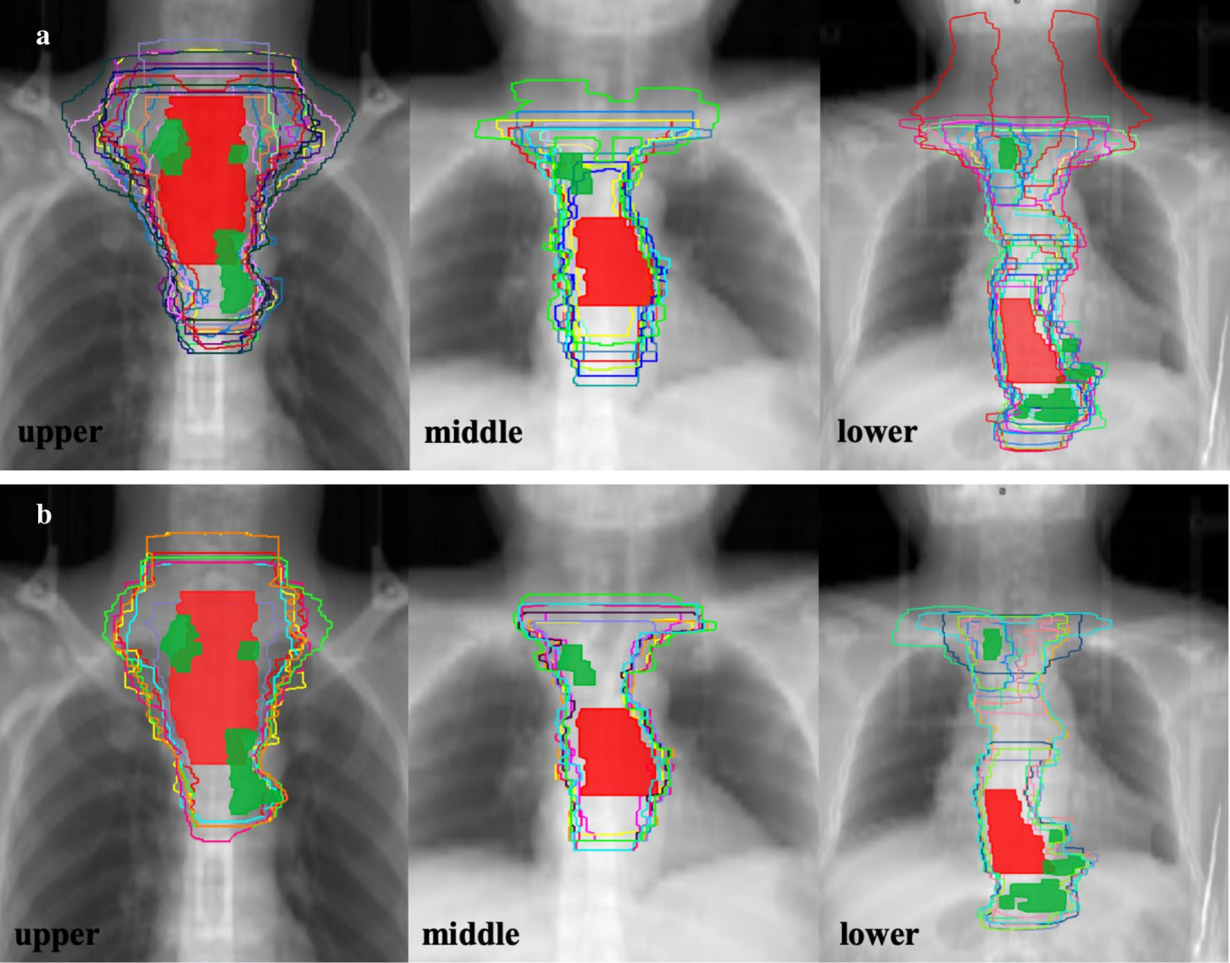
The routine group (RG; **a**) of clinical target volumes (CTVs) from 16 clinicians and the protocol group (PG; **b**) of CTVs from nine clinicians projected on one digitally reconstructed radiograph (DDR) of a CT dataset. Red and green areas indicate the primary tumor and lymph nodes, respectively

A total of 16 datasets was retrieved from 15 branch-centers in Phase 1, and one of the branch-centers included two radiation oncologists delineating target volumes separately. CTVs delineation in the RG was presented in Fig. 3a. In all three cases, the RG and PG were available from 10 clinicians; however, one of them did not comply with the protocol for the second delineation. Three participating clinicians returned their agreement on RTVs instead of contouring the new one, and the other three clinicians did not submit the protocol group of target volumes before the deadline. Figure 3b shows the CTVs in the PG, and a total of nine pairs of target volumes were included into the following comparison analysis.

### Interobserver variability

According to the RG, the result of IOV in routine clinical practice was shown in Table 1. The maximum volume of CTV in case 3 was nearly seven times that of the smallest (range, 95.9–652.9 cc) volume. In general, GTV-T showed a higher degree of consistency, of which the DSC > 0.75 in all three cases. In contrast, variability in GTV-N was larger, of which the DSC < 0.55.

**Table 1:** Interobserver variability (IOV) among 16 centers in routine clinical practice

	Volume (cc)	Dice similarity coefficient
	Mean (smallest-largest)	Mean (95% CI)
Case 1		
GTV-T	101.2 (74.7–127.7)	0.86 (0.83–0.88)
GTV-N	12.9 (4.3–30.5)	0.50 (0.41–0.58)
CTV	333.8 (204.4–442.0)	0.80 (0.76–0.82)
Case 2		
GTV-T	27.3 (21.8–34.2)	0.81 (0.79–0.83)
GTV-N^a^	2.2 (0–19.4)	0.26 (0.08–0.43)
CTV	179.8 (94.7–307.5)	0.74 (0.68–0.79)
Case 3		
GTV-T	37.9 (30.1–50.8)	0.79 (0.76–0.82)
GTV-N^a^	11.8 (5.7–32.3)^a^	0.39 (0.34–0.43)
CTV	367.6 (95.9–652.9)	0.65 (0.57–0.73)

*CI* confidence interval

^*^GTV-Ns do not conform to normal distribution as per the Shapiro–Wilk test, so the median is used instead of the mean

### Efficiency of protocol

Detailed results of the comparison between the paired groups are presented in Table 2. The use of protocol had almost improved the DSC of all target volumes, and the most significant improvement was in GTV-N, which increased from 0.51, 0.38, and 0.37 to 0.67 (*P* = 0.022), 0.55(*P* = 0.260), and 0.72 (*P* = 0.005) in case 1, case 2, and case 3, respectively. In addition, it could be observed that the CTV of case 3 had a significantly better consistency with its DSC increasing from 0.63 to 0.72 (*P* = 0.004).

**Table 2:** Interobserver variation between the routine and protocol groups from nine clinicians

	Dice similarity coefficient (95% CI)		
	Routine	Protocol	*P*
Case 1			
GTV-T	0.86 (0.82–0.89)	0.91 (0.86–0.96)	.013
GTV-N	0.51 (0.39–0.62)	0.67 (0.53–0.80)	.022
CTV	0.83 (0.79–0.86)	0.87 (0.84–0.89)	.077
Case 2			
GTV-T	0.81 (0.79–0.84)	0.83 (0.78–0.89)	.130
GTV-N^a^	0.38 (0.18–0.59)	0.55 (0.39–0.71)	.260
CTV	0.74 (0.66–0.83)	0.79 (0.75–0.84)	.084
Case 3			
GTV-T	0.79 (0.75–0.84)	0.84 (0.78–0.91)	.190
GTV-N	0.37 (0.32–0.42)	0.64 (0.45–0.83)	.005
CTV	0.63 (0.53–0.74)	0.72 (0.62–0.81)	.004

*CI* confidence interval

^a^GTV-N paired Mann–Whitney *U* test

## Discussion

In the era of precision radiotherapy, the accuracy of target volume delineation plays a significant role in planning and execution of radiotherapy. However, owing to variance in the location of primary lesions and the range of lymph node metastasis, there is variability among radiation oncologists and radiation centers with respect to in the target volumes of dRT, which may lead to delineation bias in multicenter research studies. Therefore, it is important to ensure the consistency of target volume delineation before conducting a prospective, multi-center study. Our study found that IOV existed in routine delineating practice, and the contouring protocol could help in improving the contouring consistency.

According to the RG data, the consistency in delineation of GTV-T is generally high with basic DSC above 0.75; no obvious IOV existed among clinicians and centers regardless of the location of the primary lesion. Similar results were reported by a QA program of PRODIGE 26/CONCORDE phase 2/3 trial [15] that the GTV delineation was almost respected in all centers. As also reported by Nowee et al. [16], GTV delineation consistency seemed difficult to further improved.

For GTV-N, although PET-CT, EUS, and other auxiliary examinations were provided to help diagnose metastatic lymph nodes, and the contouring protocol was applied to improve its consistency in diagnosis, its DSC value was generally lower than 0.70. The possible reasons for these results are: first, there is no clear standardized definition of metastatic lymph nodes in esophageal cancer, which may have resulted in instances of either missed or over contoured GTV-N. Second, some clinical studies [17–19] have shown that a large proportion of metastatic lymph nodes diagnosed by preoperative imaging is clinically over- or under-estimated compared with the postoperative pathological results. As reported by Mantziari et al. [20] in a study of 193 patients with esophageal cancer (clinical stage: T3N0), though the patients were enrolled into the single surgery group, pathological N0 cases accounted for only 35.8%, which indicated that more than 60% cases were under-estimated in the clinical assessment. Finally, according to the analysis by Gockel [21], there was a 27–55% rate of lymph node metastasis when the primary lesion invades the submucosa. In addition, the lymph node metastasis in esophageal cancer is very extensive. For the cases receiving three-field lymph node dissection, as reported by Isono [22], the rate of metastases in cervical nodes was 27.5% among patients with middle thoracic esophageal cancer. Therefore, it is challenging to assess lymph node metastasis in clinical practice. We reviewed the multi-center target volumes and found that variance in GTV-N is mainly due to the first reason, that is, clinicians’ cautious overestimation of suspected metastasis in lymph nodes. In reality, the introduction of protocol allows clinicians to comprehensively combine multiple diagnostic methods for judgment, which may be the reason for the increased consistency of GTV-N.

The CTV field mainly relies on clinical examinations that mainly serves to provide a reference for clinicians by improving the accuracy of judgment of metastatic lymph nodes and determination of the range of radiation treatment. In the RG, IOV in CTV were observed among branch-centers. However, for those cases with relatively limited and proven range of lymph node metastasis, the IOV was relatively small regardless of the study group, which indicates that radiation oncologists reached an agreement in delineation of such target volumes. However, in case 3, with relatively more extensive lymph node metastasis, the IOV in target volume delineation becomes an issue. The efficacy of involved field irradiation (IFI) versus elective nodal irradiation (ENI) is still debatable [23–25], and a meta-analysis [26] shows that there is no survival difference between IFI and ENI. Thus, more prospective, comparative studies should be conducted for validation. Our study suggests that regardless of the location of the primary lesion, the consistency of delineating CTV was significantly improved according to the requirements of the protocol. Therefore, the findings of this multi-center study are important because it emphasizes that a different center could achieve a more consistent target volume delineation.

A similar observation has been documented for other tumors such as nasopharyngeal, cervical, pulmonary, and gastric carcinomas [27–30]. Factors accounting for the variance in GTV-N and CTV definition in this study were similar to those found by Weiss et al. [31] who suggested that causes are multifactorial, including image- and observer-related factors. A previous study [10] suggested that refined contouring guidelines should be provided to better reduce the IOV. Accordingly, the present protocol proposed a consensus on involved lymph nodes and strict definition in the expansion criteria of CTV, leading to higher consistency in delineation of GTV-N and CTV. According to an investigation on head and neck cancers by Peters et al. [32], protocol compliance did improve the radiotherapy quality assurance to achieve optimal treatment outcomes in the combined modality (chemoradiotherapy) treatment.

To our best knowledge, this is a minority of multicenter study of the variability in the target volumes of dRT for thoracic esophageal cancer, including 16 centers throughout China. Besides, we investigated IOV with respect to both volume and spatial relationship. In addition, unlike the previous trials that included dummy runs for QA analysis [33, 34], our study showed, similar to Spoelstra et al. [28] that IOV both before and after using the protocol was used to evaluate its efficiency.

Our study explored the variability in target volumes of dRT for thoracic esophageal cancer and compensated for the gap in this field. Furthermore, our study enforced that a contouring protocol could contribute to the consistency of target volumes, making the results of multi-center studies more reliable. Except the protocol, the improvement in the contouring consistency depends on advances in diagnostics. The department of imaging diagnosis in our center indicated that combining both the lymph node size and axial ratio relationship could improve the sensitivity in diagnosis [35]. Besides, the addition of PET-CT and EUS will further increase the accuracy of N-stage [36, 37], thereby improving the consistency of target volumes definition.

One limitation of our study is that the results were based on the combination of multiple modality examinations, while patients could only receive several of them in clinical practice, which could result in more striking contouring variance. In addition, we did not ask participating centers to design treatment plans for their target volumes, and therefore variances in dosimetric parameters could not be evaluated. Instead, we planned to further assess the variability in treatment plans designed for dRT of esophageal cancer and evaluate the impact of the dose-restriction protocol on dose–volume histogram parameters.

## Conclusion

The IOV was observed in target volume delineation, and no available uniform consensus may account for it, which likely illustrates the different contouring philosophies of the participating centers and emphasizes the need for standardization. The consistency of target volumes delineation in different centers could be improved through mandatory procedure, to lay a solid foundation for the reliability of multi-center prospective studies.

## Supplementary information

**Additional file 1**. The contouring protocol for guiding the determination of target volumes.

## Data Availability

All data generated or analyzed during this study are included in this published article and its supplementary information data.

## Abbreviations

dRT: Definitive radiotherapy
IMRT: Intensity-modulated radiation therapy
SIB: Simultaneously integrated boost
GTV-T: Gross tumor volume
GTV-N: Metastatic regional nodes
IOV: Interobserver variability
OAR: Organ at risk
EGD: Esophagogastroduodenoscopy
EUS: Endoscopic ultrasound
CT: Computed tomography
PET: Positron emission tomography
CTV: Clinical target volume
RG: Routine group
RTV: Referential target volume
PG: Protocol group
DSC: Dice similarity coefficient
IFI: Involved field irradiation
ENI: Elective nodal irradiation
