# Supplementary material for: Interobserver variability in target volume delineation in definitive radiotherapy for thoracic esophageal cancer: a multi-center study from China
Source: Radiat Oncol. 2021 Jun 9;16:102. doi: 10.1186/s13014-020-01691-4 (PMC8188796; doi:10.1186/s13014-020-01691-4)
Supplement: Supplementary file 1 — Additional file 1. The contouring protocol for guiding the determination of target volumes. [file 13014_2020_1691_MOESM1_ESM.doc]

Additional file 1: Appendix A

Contouring Protocol

1. The primary lesion should be identified by available pre-treatment information such as symptoms, physical examination, and radiology and endoscopy results and, if possible, the planning computed tomography (CT) is recommended to be fused with planning positron emission tomography computed tomography (PET/CT) or magnetic resonance imaging (MRI) before delineation.
2. The diagnostic criteria for involved lymph nodes are as follows: First, metastatic lymph nodes are clearly diagnosed by pre-treatment diagnostic examinations including supraclavicular ultrasonic examination, endoscopic ultrasound (EUS), contrast-enhanced CT, PET/CT; Second, the highly suspicious lymph nodes could not be excluded in clinical practice; Third, according to clinical experience, if the thoracic para-esophageal lymph nodes were in the round shape, without the normal lymphatic hilum or larger than 5 mm in short-axis diameter at the same time, these highly suspicious lymph nodes could also be considered as metastatic;
3. The craniocaudal expansion criteria for clinical target volume (CTV) from a primary lesion are as follows: In the first situation, if the primary lesion with a 3.0-cm craniocaudal margin could cover all suspicious lymph node regions within appropriate subclinical margins, then a 3-cm expansion was acceptable. In the second situation, if there are some highly suspicious lymph nodes that could not be ignored within the region between 3 and 5 cm of the craniocaudal expansion margin from the primary lesion, the 5-cm expansion is highly recommended. It is noticed that the 5-cm expansion is not required in both cranial and caudal directions and thus a unilateral expansion makes sense. In the third situation, if the primary lesion with a 5-cm craniocaudal margin could not include involved lymph nodes, then a 3-cm expansion from the primary lesion and 1-cm expansion from the lymph nodes was acceptable. This situation could be observed in cases of upper thoracic esophageal cancer with abdominal lymph node metastasis or the lower thoracic esophageal cancer with upper mediastinal lymph node metastasis.
